# Preoperative predictors of difficult laparoscopic cholecystectomy after percutaneous transhepatic gallbladder drainage in patients with cholecystitis

**DOI:** 10.1016/j.clinsp.2026.101132

**Published:** 2026-09-09

**Authors:** Chaoyi Zhou, Zeliang Xia, Liang Chu, Zhaolong Xu, Zhengmin Chen

**Affiliations:** Department of General Surgery, The Second Affiliated Hospital of Bengbu Medical University, Bengbu, Anhui, China

**Keywords:** Laparoscopic cholecystectomy, Percutaneous transhepatic gallbladder drainage, Surgical difficulty prediction, Inflammatory biomarkers, Nomogram

## Abstract

**Background:**

Percutaneous Transhepatic Gallbladder Drainage (PTGBD) serves as a bridge therapy for moderate-to-severe cholecystitis. However, Laparoscopic Cholecystectomy (LC) after PTGBD is frequently complicated by chronic inflammation and fibrosis. Currently, predictive tools for difficult surgery in this population are limited, and none have integrated multidimensional parameters including novel imaging metrics and psychological assessments.

**Methods:**

We retrospectively analyzed 150 patients who underwent LC after PTGBD between September 2018 and May 2025. After excluding 43 patients (22 with missing data, 21 with combined procedures), 107 were included. Using univariate and multivariate logistic regression, we developed a nomogram incorporating five-dimensional preoperative parameters: 1) Baseline clinical characteristics, 2) CT imaging features, 3) PTGBD-related surgical parameters, 4) Laboratory indicators, and 5) Psychological assessments. Difficult surgery was defined by Tokyo Guidelines (TG18) criteria: operative time > 110 min, blood loss > 150 mL, or conversion to open surgery (including subtotal cholecystectomy or fundus-first technique).

**Results:**

Multivariate analysis identified four independent predictors: elevated C-reactive protein (CRP > 8.2 mg/L; OR = 4.21, p = 0.011), pericholecystic fluid (OR = 60.93, p < 0.001), gallbladder-duodenal adhesion index (GDAI > 0.7; OR = 4.00, p = 0.015), and increased Calot's triangle CT attenuation (HU > 30; OR = 5.82, p < 0.001). The nomogram demonstrated excellent discrimination (AUC = 0.903), significantly outperforming individual predictors (p < 0.001).

**Conclusions:**

The model enables precise risk stratification, which could potentially guide clinical decisions and improve surgical outcomes.

## Highlights

• First prediction model for difficult LC after PTGBD.
• Four independent predictors: CRP, PCF, GDAI, HU.
• Nomogram shows excellent discrimination (AUC = 0.903).
• Guides surgeon selection and adhesion preparedness.
• Integrates inflammation and local anatomical changes.

## Introduction

Laparoscopic Cholecystectomy (LC) is the gold standard for cholecystitis treatment. For patients with moderate-to-severe cholecystitis unfit for immediate surgery, Percutaneous Transhepatic Gallbladder Drainage (PTGBD) provides critical bridging therapy by reducing intraluminal pressure and controlling sepsis.^1^ However, PTGBD-treated patients often develop chronic inflammation and fibrosis, leading to challenging LC characterized by dense adhesions in Calot's triangle, obscured anatomy, and bleeding risks. These complexities increase operative time, conversion rates, complications, and healthcare costs.^2^

Existing prediction models for difficult LC focus primarily on acute cholecystitis and lack applicability to PTGBD-treated populations due to unique pathophysiological changes: indwelling catheters may accelerate fibrosis, while chronic inflammation promotes adhesion formation. Although TG18 defines objective criteria for difficult LC, preoperative prediction remains challenging.^3^^,^^4^ Current models rely on isolated parameters (e.g., age, leukocyte count, gallbladder wall thickness) without integrating multidimensional data (imaging features, dynamic inflammatory changes, comorbidities).^5^ Emerging evidence suggests psychological factors may influence surgical outcomes through neuroendocrine pathways, but their role in PTGBD-LC difficulty prediction is unexplored.^6^

Computed Tomography (CT)-based imaging features show promise for surgical difficulty assessment.^7^ Increased Hounsfield Units (HU) in Calot's triangle indicate fat stranding/fibrosis, Pericholecystic Fluid (PCF) reflects inflammation severity, and the Gallbladder-Duodenal Adhesion Index (GDAI) quantifies adhesions.^8^, ^9^, ^10^ Systemic inflammatory markers like CRP, Neutrophil-Lymphocyte Ratio (NLR), and Transforming Growth Factor-β1 (TGF-β1) correlate with fibrosis but lack validated integration into predictive tools.^11^, ^12^, ^13^

This study aims to develop the first comprehensive prediction model for difficult LC after PTGBD by analyzing five-dimensional preoperative parameters: 1) Baseline clinical characteristics and comorbidities (age, BMI, TG18 grading, CCI, ASA), 2) CT imaging features (Calot's triangle HU, PCF, GDAI, gallbladder morphology), 3) PTGBD-related surgical parameters (catheter status, PTGBD-to-LC interval), 4) Laboratory indicators (CRP, NLR, TGF-β1, albumin, ALT, WBC), and 5) Psychological assessments (HADS-A and HADS-D). The resulting nomogram will facilitate individualized risk assessment and optimize clinical decision-making.

## Materials and methods

### Study population

This retrospective study included cholecystitis patients who underwent LC after PTGBD at our institution (09/2018–05/2025). Exclusion criteria: 1) Combined procedures (e.g., hepatic cyst fenestration, common bile duct exploration), 2) Missing > 20% of core variables (CRP, CT parameters, operative records). 3) Acalculous cholecystitis. All included patients had calculous cholecystitis confirmed by preoperative imaging and intraoperative findings. Patients with acalculous cholecystitis were excluded from this study due to its distinct pathophysiology and potentially different impact on surgical difficulty. All surgical procedures were conducted by surgeons with advanced expertise in laparoscopic techniques. Ethical clearance for this retrospective analysis was granted by the institutional review board (Approval n° 2018,011). Written informed consent was obtained from all patients for the publication of any potentially identifiable images or data included in this article.

### Data collection

Through structured electronic medical records, we collected five categories of parameters: 1) Baseline clinical characteristics: including gender, age, Body Mass Index (BMI), Tokyo Guidelines 2018 (TG18)-based cholecystitis grading, Charlson Comorbidity Index (CCI), ASA grade, abdominal surgical history, and presence of cirrhosis, and number of previous cholecystitis episodes (defined as documented episodes of acute cholecystitis prior to the current admission). 2) Imaging data: All patients underwent abdominal CT 24-hours before LC, which was assessed by two blinded radiologists for the average CT value (standardized 10 mm^2^ region of interest) of Calot's triangle, abnormal gallbladder wall (thickness > 4 mm or roughness), pericholecystic fluid, gallbladder-duodenal adhesion index (GDAI; contact area ratio between gallbladder and duodenum on 3D CT reconstruction, using GE AW4.7 workstation with 1 mm slice thickness arterial/portal venous phase images). GDAI = (gallbladder-duodenal contact area / total gallbladder surface area), with GDAI ≥ 0.7 indicating significant adhesion. Stone characteristics (maximum diameter/number/location). Impacted gallstones were defined as stones lodged in the gallbladder neck or cystic duct causing obstruction on preoperative CT. 3) Surgical parameters: PTGBD catheter removal, interval time between PTGBD and LC (defined as days from successful PTGBD to LC), surgical time, conversion to open surgery (including subtotal cholecystectomy or fundus-first (antegrade) cholecystectomy), and intraoperative blood loss were recorded. The interval between symptom onset and PTGBD (in days) was recorded for all patients. 4) Laboratory indicators: Inflammatory and hepatic indicators were detected within 72-hours before LC, including CRP, WBC, NLR, TGF-β1, albumin, and ALT, with abnormal thresholds strictly defined (e.g., CRP > 8.2 mg/L, NLR > 3.5). These thresholds (CRP ≥ 8.2 mg/L, NLR ≥ 3.5, WBC ≥ 9 × 10⁹/L, TGF-β1 ≥ 40 ng/mL) were derived from ROC curve analysis maximizing Youden's index for association with difficult surgery in this cohort. 5) Psychological assessment parameters: Psychological status was evaluated preoperatively using the Hospital Anxiety and Depression Scale (HADS), administered to all participants 24–72 hours prior to surgery. This validated instrument contains 14-items subdivided into anxiety (7-items) and depression (7-items) domains, each rated on a 4-point Likert scale (0–3). According to Zigmond's criteria, a subscale score ≥ 8 indicates positive anxiety/depression. Trained nurses guided the completion in a quiet environment. Postoperative outcomes including hospital stay, total cost, and Clavien-Dindo ≥ Grade II complications were also recorded.

Laboratory tests were conducted within 3-days preoperatively, and CT scans were performed within 1-day preoperatively. In this study, abnormal thresholds were defined as follows: elevated CRP was defined as CRP > 8.2 mg/L; elevated WBC as WBC > 9 × 10⁹/L; hypoalbuminemia as albumin < 35 g/L; elevated ALT as ALT > 40 U/L; elevated NLR as NLR > 3.5; elevated TGF-β1 as TGF-β1 > 40 ng/mL; elevated Calot's triangle CT-value as HU > 30; and significant gallbladder-duodenal adhesion as a gallbladder-duodenal adhesion index > 0.7.

### PTGBD indications

According to the Tokyo Guidelines 2018 (TG18), PTGBD was indicated in patients with acute cholecystitis who were not suitable for immediate laparoscopic cholecystectomy. In our study, the indications for PTGBD were as follows: 1) Grade II (moderate) acute cholecystitis with high surgical risk (Charlson Comorbidity Index ≥6 and/or ASA class ≥ III), n = 98; 2) Grade III (severe) acute cholecystitis requiring urgent biliary decompression after initial medical stabilization, n = 9.

No patients with Grade I (mild) acute cholecystitis underwent PTGBD.^14^ It should be emphasized that PTGBD was not performed solely based on ASA class; in patients with Grade II acute cholecystitis, the decision reflected an individualized assessment of overall surgical risk, comorbidity burden, and response to initial conservative management, rather than early disease duration alone.

### Definition of difficult surgery

While standardized criteria for surgical difficulty remain elusive, the Tokyo Guidelines 2018 (TG18) identify operative duration, conversion rates, and complications as key determinants of challenging LC. Aligned with these parameters and absent intraoperative complications in our cohort, cases fulfilling ≥ 1 of the following objective thresholds were designated as difficult: 1) Procedure time exceeding 110-minutes; 2) Intraoperative hemorrhage > 150 mL; 3) Necessity for bail-out procedures, including fundus-first dissection, subtotal cholecystectomy, or conversion to open surgery.^15^, ^16^, ^17^, ^18^

### Treatment strategy

Managed according to TG18 grading: Grade I cholecystitis: Early LC within 7-days of onset. Grade II cholecystitis: Antibiotics → if failed, emergency PTGBD → deferred LC; if inflammation is controlled, early LC (CCI ≤5 and ASA≤II) or deferred LC (CCI≥6 or ASA≥III) is selected based on CCI/ASA. Grade III cholecystitis: Organ support + antibiotics → for patients with high-risk factors (jaundice/neurological impairment/respiratory failure), emergency PTGBD is performed, followed by assessment for deferred LC (CCI≤3 or ASA≤2) or non-surgical treatment (CCI≥4 and ASA≥ 3).

### Statistical analysis

Categorical data are reported as counts (percentages) and compared using χ^2^ or Fisher's exact tests. Continuous variables conforming to normality assumptions are presented as mean ± standard deviation and analyzed with Student's *t*-tests; non-parametric data are summarized as median (interquartile range) and assessed via Mann-Whitney *U tests*. Optimal cut-off values for continuous variables (e.g., CRP, NLR) were determined by ROC curve analysis based on the Youden index, as detailed in the Data Collection section. Potential predictors (p < 0.1 in univariate logistic regression) underwent multivariate logistic regression with backward elimination to identify independent risk factors. A nomogram integrating these factors was developed and evaluated through Receiver Operating Characteristic (ROC) analysis (Area Under the Curve, AUC), Hosmer-Lemeshow calibration, and Decision Curve Analysis (DCA). Statistical significance was defined as two-tailed p < 0.05. Analyses utilized SPSS 24.0 and R 4.2.2 (employing "rms", "pROC", and "rmda" packages). p-values in Table 1 are derived from unadjusted group comparison tests, whereas p-values in Table 2 originate from univariate logistic regression models; therefore, numerical differences between these p-values are expected and reflect methodological differences rather than inconsistencies in the data.

**Table 1:** Preoperative baseline clinical, laboratory, and imaging characteristics of patients undergoing LC after PTGBD.

Factor	Non-Difficult (n = 77)	Difficult (n = 30)	p-value
Demographics			
Sex			0.827
Female	41 (53.2%)	17 (56.7%)	
Male	36 (46.8%)	13 (43.3%)	
BMI (kg/m²)	24.8 (2.3)	25.3 (1.7)	0.520
Age (years)			0.751
< 60	26 (33.8%)	11 (36.7%)	
≥ 60	51 (66.2%)	19 (63.3%)	
Cholecystitis severity			0.720
Grade II	70 (90.9%)	28 (93.3%)	
Grade III	7 (9.1%)	2 (6.7%)	
Cirrhosis, n (%)	2 (2.6%)	1 (3.3%)	0.567
Number of previous cholecystitis episodes, median (IQR)	1 (1‒2)	2 (1‒3)	0.032
Impacted gallstones, n (%)	11 (14.3%)	8 (26.7%)	0.132
CCI (points)	2 (1)	3 (2)	0.001
ASA> 3	16 (20.8%)	9 (30.0%)	0.501
Prior abdominal surgery	15 (19.5%)	8 (26.7%)	0.433
Imaging			
Calot's triangle HU > 30	14 (18.2%)	22 (73.3%)	<0.001
Maximal stone diameter (cm)	0.4 (1.6)	0.5 (1.5)	0.417
Pericholecystic Fluid (PCF)	2 (2.6%)	15 (50.0%)	<0.001
Multiple stones	21 (27.3%)	11 (36.7%)	0.570
Gallbladder-duodenal adhesion index (GDAI) > 0.7	36 (46.8%)	23 (76.7%)	0.006
Infundibular stone	12 (15.6%)	8 (26.7%)	0.242
Abnormal gallbladder wall	13 (16.9%)	12 (40.0%)	0.040
PTGBD parameters			
Catheter removal	22 (28.6%)	8 (26.7%)	0.756
PTGBD-to-LC interval (days)	55 (47)	46.5 (50)	0.631
Laboratory			
NLR > 3.5	12 (15.6%)	18 (60.0%)	<0.001
CRP > 8.2 mg/L	16 (20.8%)	14 (46.7%)	0.027
TGF-β1 > 40 ng/mL	10 (13.0%)	15 (50.0%)	<0.001
Albumin < 35 g/L	3 (3.9%)	5 (16.7%)	0.088
ALT > 40 U/L	12 (15.6%)	6 (20.0%)	0.572
WBC > 9 × 10⁹/L	3 (3.9%)	5 (16.7%)	0.088

Note: HU, Hounsfield Units; LC, Laparoscopic Cholecystectomy; PTGBD, Percutaneous Transhepatic Gallbladder Drainage; BMI, Body Mass Index; TG18, Tokyo Guidelines 2018; CCI, Charlson Comorbidity Index; ASA, American Society of Anesthesiologists; HU, Hounsfield Units; PCF, Pericholecystic Fluid; GDAI, Gallbladder-Duodenal Adhesion Index; CRP, C-Reactive Protein; WBC, White Blood Cell Count; NLR, Neutrophil-to-Lymphocyte Ratio; TGF-β1, Transforming Growth Factor beta-1; HADS, Hospital Anxiety and Depression Scale.

**Table 2:** Univariate logistic regression analysis of preoperative factors associated with difficult LC.

Factor	OR (95% CI)	p-value
CCI	1.25 (1.04–1.51)	0.019
Elevated CRP	3.10 (1.26–7.62)	0.013
Elevated WBC	3.00 (1.11–8.10)	0.030
Hypoalbuminemia	2.95 (1.11–7.85)	0.030
Number of previous cholecystitis episodes	1.42 (1.03‒1.96)	0.032
Tokyo Grade III (vs. II)	1.45 (0.52‒4.03)	0.478
Pericholecystic Fluid (PCF)	5.20 (2.30–11.76)	<0.001
GDAI > 0.7	8.24 (3.52–19.30)	<0.001
Abnormal gallbladder wall	3.50 (1.15–10.65)	0.028
Calot's triangle HU > 30	12.86 (4.82–34.32)	<0.001
NLR > 3.5	8.42 (3.28–21.62)	<0.001
TGF-β1 > 40 ng/mL	6.75 (2.61–17.45)	<0.001

Note: Included for clinical relevance despite non-significance.

## Results

### Patient characteristics

**Fig. 1:**
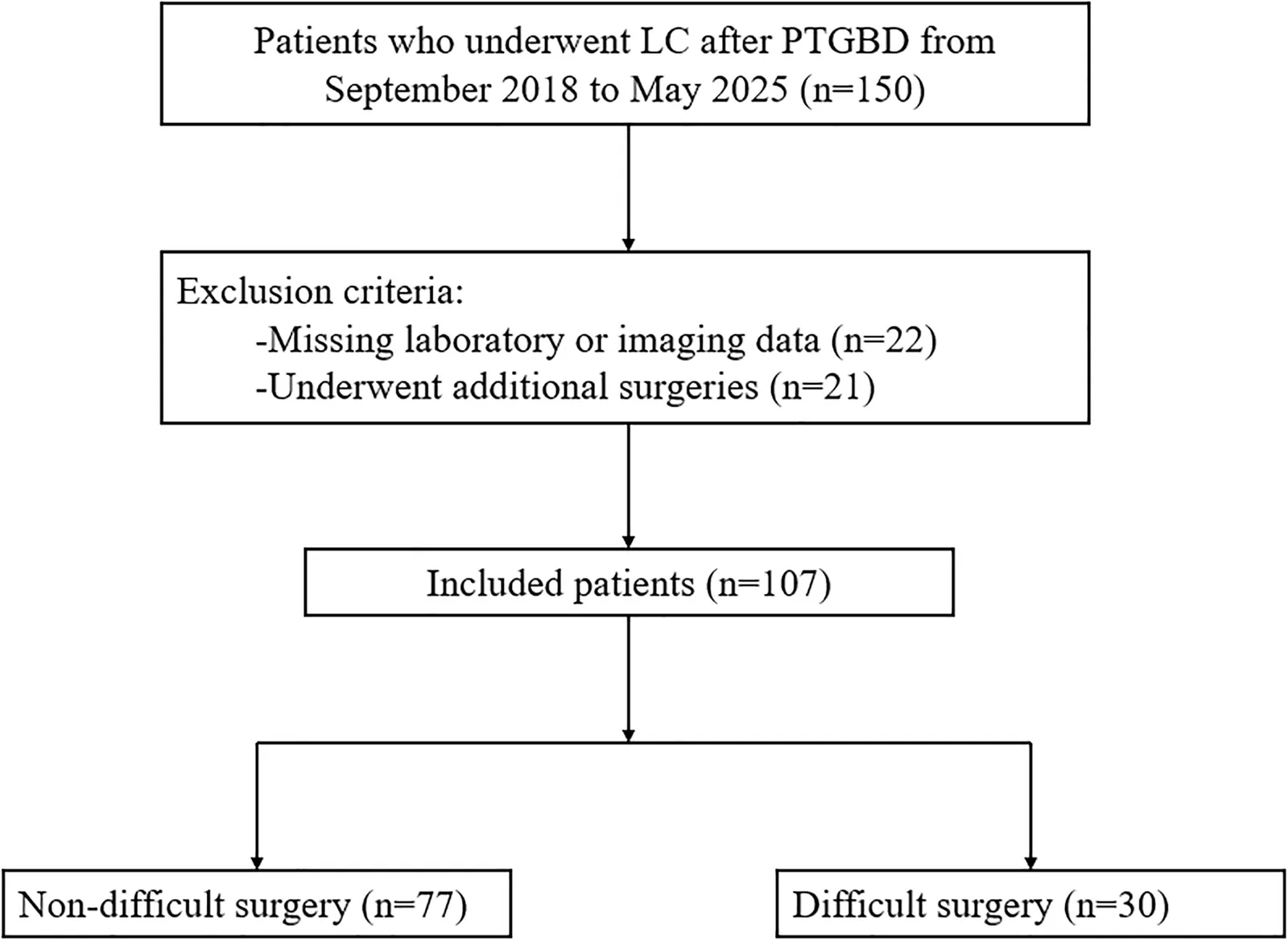
Patient selection flowchart.

Among 150 screened patients, 22 with missing data and 21 with combined procedures were excluded. The final cohort included 107 patients: difficult surgery group (n = 30, 28.0%), non-difficult surgery group (n = 77, 72.0%; Fig. 1).

Table 1 compares preoperative characteristics. No significant differences existed in sex, age, BMI, cholecystitis severity, ASA classification, or prior abdominal surgery (all p > 0.05). The prevalence of cirrhosis was low and comparable between groups (2.6%vs. 3.3%, p = 0.567). The difficult surgery group had a higher median number of previous cholecystitis episodes [2 (IQR 1‒3) vs. 1 (IQR 1‒2), p = 0.032]. The incidence of impacted gallstones was higher in the difficult group (26.7%vs. 14.3%), but this difference did not reach statistical significance (p = 0.132). The difficult surgery group had higher CCI scores (3[2]) vs. 2[1] points, p = 0.001). Laboratory results showed higher rates of elevated NLR (60.0%vs. 15.6%, p < 0.001), CRP (46.7%vs. 20.8%, p = 0.027), and TGF-β1 (50.0%vs. 13.0%, p < 0.001) in the difficult group. Hypoalbuminemia and leukocytosis showed non-significant trends (p = 0.088). Imaging revealed more frequent PCF (50.0%vs. 2.6%, p < 0.001), elevated GDAI (76.7%vs. 46.8%, p = 0.006), abnormal gallbladder wall morphology (40.0%vs. 16.9%, p = 0.040), and increased Calot's triangle HU (73.3%vs. 18.2%, p < 0.001) in the difficult group. Stone characteristics and PTGBD-related parameters showed no differences (all p > 0.05). The median PTGBD-to-LC interval was 55 (47) days in the non-difficult group and 46.5 (50) days in the difficult group, with no significant difference between groups (p = 0.631).

Pre-PTGBD laboratory values (Table 3) showed no significant intergroup differences (all p > 0.05), though WBC, NLR, and TGF-β1 were numerically higher in the difficult group. The time from symptom onset to PTGBD was similar between groups (median 3 vs. 4-days, p = 0.241). Operative outcomes (Table 4): The difficult surgery group had longer operative time (105.5 vs. 84 min, p < 0.001), higher conversion rate (13.33%vs. 0%, p = 0.011), longer hospitalization (6 vs. 5 days, p < 0.001), and higher costs ($4143 ± $1090 vs. $3626 ± $921, p < 0.001). Although median blood loss was identical between groups (25 mL in both), the difficult surgery group exhibited significantly greater variability in blood loss (IQR: 45 vs. 15 mL, p < 0.001 by Mann-Whitney *U test*), reflecting more frequent episodes of clinically significant bleeding in the upper quartile. Clavien-Dindo ≥ Grade II complications were more frequent in the difficult group (16.67%vs. 1.30%, p = 0.039). Clavien-Dindo Grade ≥II complications occurred in 1-patient (1.3%) in the non-difficult group (surgical site infection, Grade II) and in 6-patients (16.7%) in the difficult group, including surgical site infection (n = 2, Grade II), intra-abdominal abscess requiring percutaneous drainage (n = 2, Grade IIIa), postoperative bleeding requiring intervention (n = 1, Grade IIIa), and bile leak requiring ERCP (n = 1, Grade IIIa). Notably, there were no cases of iatrogenic bile duct injury in either group (0% in both). Psychological assessment (Table 5): The difficult surgery group had higher HADS-A (7.1 ± 2.8 vs. 5.2 ± 2.1, p = 0.002) and HADS-D scores (6.3 ± 2.1 vs. 4.8 ± 1.9, p = 0.007), with higher rates of anxiety (36.7%vs. 15.6%, p = 0.018) and depression (30.0%vs. 13.0%, p = 0.045).

**Table 3:** Pre-PTGBD clinical and laboratory parameters.

Parameter	Non-Difficult (n = 77)	Difficult (n = 30)	p-value
WBC (× 10⁹/L)	15.2 (7.1)	18.4 (8.9)	0.083
Hemoglobin (g/L)	131 (19)	127 (18)	0.326
Albumin (g/L)	36.0 (6.1)	33.8 (7.2)	0.138
Total bilirubin (μmoL/L)	18.5 (9.6)	24.0 (12.3)	0.054
ALT (U/L)	47.5 (27.8)	54.2 (30.1)	0.287
CRP (mg/L)	7.3 (6.3)	7.35 (4.5)	0.703
NLR	2.9 (1.1)	3.7 (1.1)	0.112
TGF-β1 (ng/mL)	30.9 (5.6)	41.65 (5.08)	0.087
Symptom onset to PTGBD (days), median (IQR)	3 (2‒5)	4 (2‒6)	0.241

Note: Values are presented as mean (standard deviation) for normally distributed variables and median (interquartile range) for non-parametric variables. p-values derived from Student's *t*-test or Mann-Whitney *U test* as appropriate.

**Table 4:** Operative characteristics and short-term postoperative outcomes.

Outcome	Non-Difficult (n = 77)	Difficult (n = 30)	p-value
Hospital costs (USD)	3626±921	4143±1090	<0.001
Hospital stay (days)	5 (3)	6 (4)	<0.001
Clavien-Dindo ≥ Grade II	1 (1.3%)	6 (16.7%)	0.039
Operative time (min)	84 (37)	105.5 (15.5)	<0.001
Blood loss (mL)	25 (15)	25 (45)	<0.001
conversion to open surgery (including subtotal cholecystectomy or fundus-first technique)	0 (0%)	4 (13.3%)	0.011
Bile duct injury, n (%)	0 (0%)	0 (0%)	1.000

Note: Complications in the difficult group: surgical site infection (n = 2), intra-abdominal abscess (n = 2), postoperative bleeding (n = 1), bile leak (n = 1). Continuous variables are presented as mean ± standard deviation or median (Interquartile Range [IQR]). p-values were calculated using Student's *t*-test for normally distributed data and the Mann-Whitney *U test* for non-parametric data.

**Table 5:** Preoperative psychological assessment using the Hospital Anxiety and Depression Scale (HADS).

Parameter	Non-Difficult (n = 77)	Difficult (n = 30)	p-value
HADS-A score (points)	5.2 ± 2.1	7.1 ± 2.8	0.002
HADS-A ≥ 8, n (%)	12 (15.6%)	11 (36.7%)	0.018
HADS-D score (points)	4.8 ± 1.9	6.3 ± 2.1	0.007
HADS-D ≥ 8, n (%)	10 (13.0%)	9 (30.0%)	0.045

### Risk factor analysis for difficult surgery

Table 2 summarizes the univariate logistic regression assessing associations between preoperative variables and surgical difficulty (inclusion threshold: p < 0.1), comparing baseline characteristics across the non-difficult and difficult cohorts. The Charlson Comorbidity Index (OR = 1.25, 95% CI 1.04‒1.51, p = 0.019), elevated CRP (OR = 3.10, 95% CI 1.26‒7.62, p = 0.013), elevated WBC (OR = 3.00, 95% CI 1.11‒8.10, p = 0.030), hypoalbuminemia (OR = 2.95, 95% CI 1.11‒7.85, p = 0.030), pericholecystic fluid (OR = 5.20, 95% CI 2.30‒11.76, p < 0.001), elevated gallbladder-duodenal adhesion index (OR = 8.24, 95% CI 3.52‒19.30, p < 0.001), abnormal gallbladder wall morphology (OR = 3.50, 95% CI 1.15‒10.65, p = 0.028), elevated Calot's triangle CT value (HU) (OR = 12.86, 95% CI 4.82‒34.32, p < 0.001), elevated NLR (OR = 8.42, 95% CI 3.28‒21.62, p < 0.001), and elevated TGF-β1 (OR = 6.75, 95% CI 2.61‒17.45, p < 0.001) were all significantly associated with difficult surgery (p < 0.05). Psychological scores differed between groups but were not independent predictors. Although the difficult surgery group had higher psychological scores, their predictive value was overshadowed by stronger imaging and inflammatory factors, indicating that biomedical indicators dominate prediction in acute inflammation.

Table 6 details the subsequent multivariate logistic regression, which incorporated significant univariate predictors to identify independent determinants of difficult LC. The results showed that elevated CRP (OR = 4.21, 95% CI 1.61‒11.81, p = 0.011), pericholecystic fluid (OR = 60.93, 95% CI 12.12‒324.16, p < 0.001), elevated gallbladder-duodenal adhesion index (OR = 4.00, 95% CI 1.31‒12.25, p = 0.015), and elevated Calot's triangle CT value (HU) (OR = 5.82, 95% CI 2.15‒15.76, p < 0.001) were independent risk factors for difficult surgery (p < 0.05), with statistical significance in predicting difficult surgery. Tokyo Grade III was not significantly associated with difficult surgery in univariate analysis (OR = 1.45, 95% CI 0.52‒4.03, p = 0.478).

**Table 6:** Multivariate logistic regression identifying independent predictors of difficult LC.

Factor	OR (95% CI)	p-value
Elevated CRP	4.21 (1.61–11.81)	0.011
Pericholecystic fluid (PCF)	60.93 (12.12–324.16)	<0.001
GDAI > 0.7	4.00 (1.31–12.25)	0.015
Calot's triangle HU > 30	5.82 (2.15–15.76)	<0.001

### Development and validation of the prediction model

**Fig. 2:**
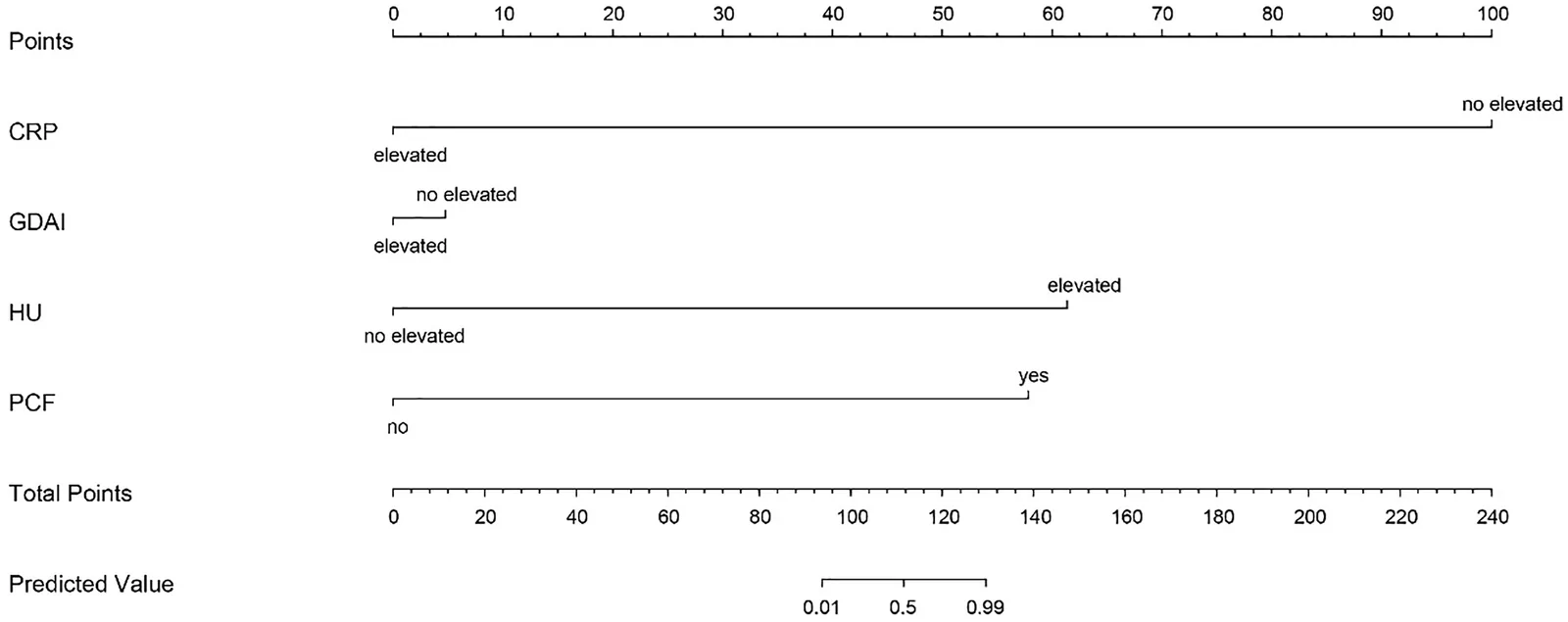
Nomogram for predicting difficult LC after PTGBD.

**Fig. 3:**
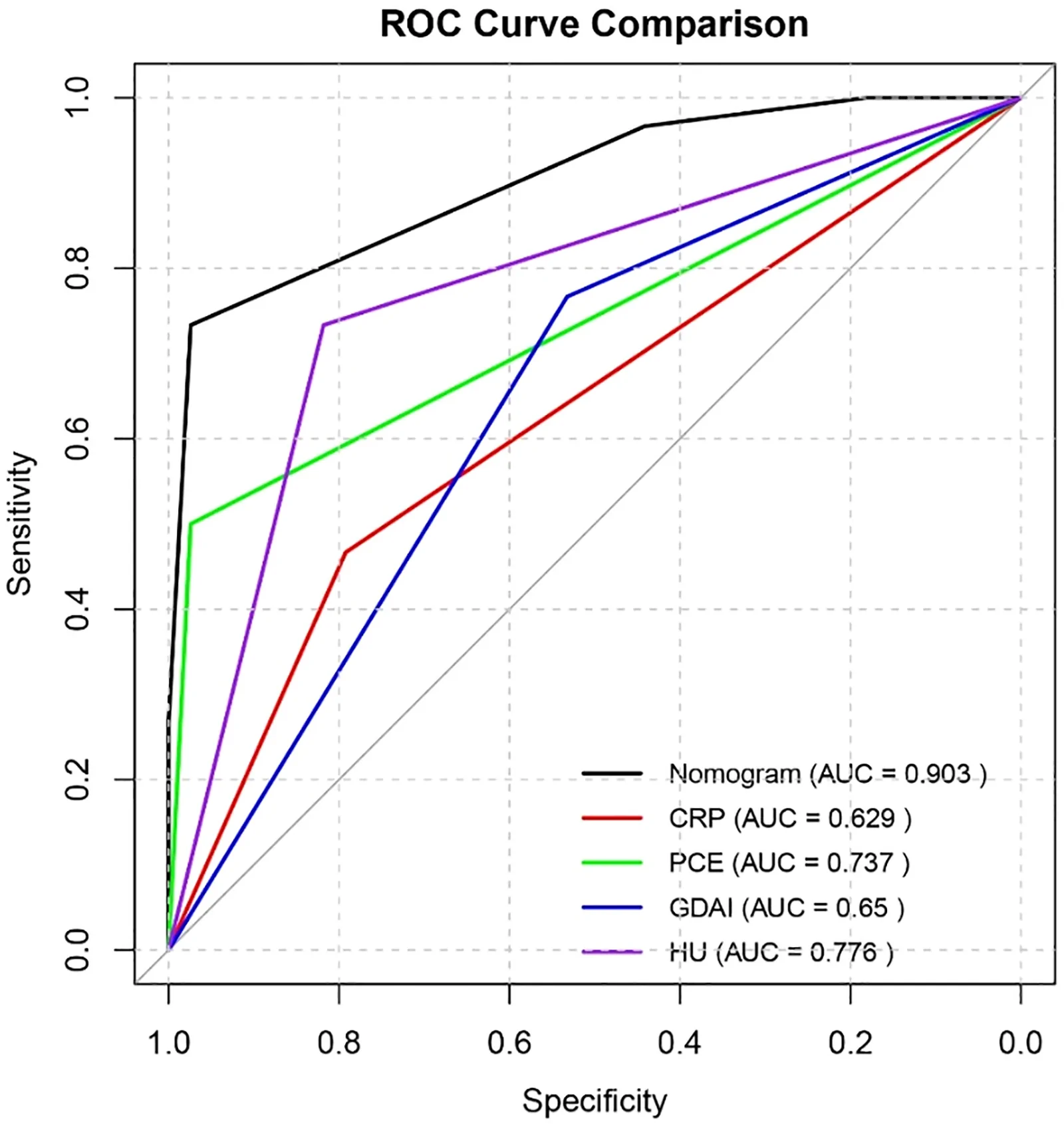
ROC curves of the nomogram and individual predictors.

**Fig. 4:**
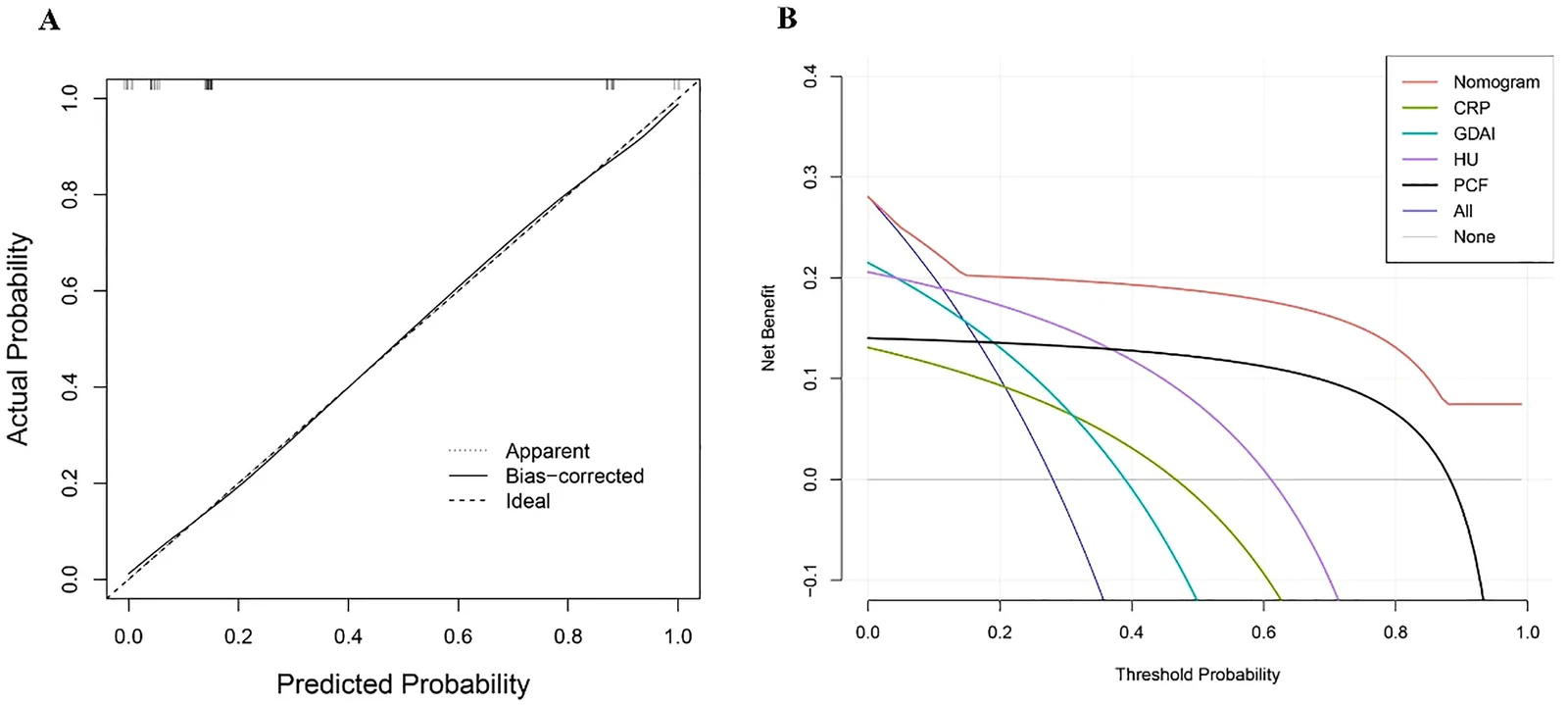
Model validation. (A) Calibration curve; (B) Decision curve analysis.

The nomogram incorporating the four independent predictors is shown in Fig. 2. It demonstrated excellent discrimination (AUC = 0.903; 95% CI: 0.842–0.964), significantly outperforming individual predictors (CRP: AUC = 0.629; GDAI: AUC = 0.650; HU: AUC = 0.776; PCF: AUC = 0.737; all p < 0.001; Fig. 3). The calibration curve demonstrated strong concordance between nomogram-predicted probabilities and actual outcomes (Fig. 4A). Decision curve analysis (Fig. 4B) further validated the model’s clinical utility, revealing significantly greater net benefit across probability thresholds relative to individual predictors or blanket intervention approaches.

The points assigned to each factor in the nomogram correspond to the predictive weight of each variable derived from multivariate logistic regression. Higher scores indicate a greater likelihood of difficult surgery. Higher points are assigned to elevated CRP, elevated GDAI, elevated Calot's triangle HU, and the presence of pericholecystic fluid, consistent with their positive associations with surgical difficulty in the multivariate model.

## Discussion

Our findings are consistent with recent studies that have identified inflammatory markers as key predictors of surgical difficulty after PTGBD. Wei et al.^1^ reported that preoperative CRP and local inflammation (gallbladder wall thickness) were independent risk factors for difficult LC, which aligns with our identification of CRP and pericholecystic fluid as significant predictors. Similarly, Lyu et al.^13^ developed a predictive model for Grade II acute cholecystitis and found that CRP and gallbladder wall thickness were important components. However, our study extends these findings by incorporating novel imaging parameters (GDAI and Calot's triangle HU) and demonstrates that these anatomical markers may have stronger predictive value than systemic inflammation alone in the multivariate model. This suggests that while systemic inflammation contributes to difficulty, the ultimate technical challenge is determined by the resulting anatomical distortion. This study establishes a comprehensive prediction model for difficult LC after PTGBD, integrating inflammatory, anatomical, and imaging biomarkers. We identified four independent predictors: elevated CRP, PCF, increased GDAI, and higher Calot's triangle HU – collectively reflecting chronic inflammation-fibrosis pathways.

Pathophysiologically, CRP elevation indicates systemic inflammation that promotes TGF-β1-mediated collagen deposition.^19^ PCF signifies transmural inflammation causing fat stranding (HU > 30) and microvascular proliferation, increasing bleeding risk during dissection.^20^ GDAI > 0.7 reflects established fibrotic adhesions distorting anatomy. Notably, while TGF-β1 and NLR correlated with difficulty in univariate analysis, they were superseded by direct anatomical markers (PCF, GDAI, HU) in multivariate modeling, emphasizing the primacy of local structural changes over systemic biomarkers for predicting technical complexity.^21^

Our findings challenge conventional LC difficulty predictors. Unlike acute cholecystitis studies where gallbladder wall thickness predicts difficulty (e.g., Noguchi et al.),^22^ neither demographic factors (age, sex, BMI) nor gallbladder morphology retained significance in PTGBD patients. We posit that catheter-induced mechanical irritation exacerbates fibrosis, diminishing the predictive value of conventional morphological features. Instead, dynamic inflammation (CRP) and spatial adhesion metrics (GDAI) better reflect chronic anatomical distortion. Although CRP is a non-specific inflammatory marker, it was included as a candidate variable based on prior evidence linking systemic inflammation to pericholecystic fibrosis; in this study, CRP dichotomization served a pragmatic modeling purpose, and its predictive relevance should be interpreted only in conjunction with local anatomical and imaging findings rather than as an isolated determinant of surgical difficulty.

Although psychological distress (anxiety/depression) was more prevalent in the difficult surgery group, it did not independently predict surgical complexity.^23^ This suggests anatomical and inflammatory factors predominantly determine intraoperative difficulty, while psychological status may influence postoperative recovery through distinct pathways.^24^ Although psychological scores showed significant between-group differences, they were not included as independent predictors in the multivariate model, as these variables did not retain significance after adjustment for objective inflammatory and imaging parameters and were therefore considered exploratory rather than causative factors of surgical difficulty.

The nomogram based on four factors (AUC = 0.903) enables a shift from “population-based” to “individualized” risk assessment. It directly informs clinical decisions: High-risk patient management: For patients with CRP > 8.2 mg/L, pericholecystic fluid on CT, GDAI > 0.7, or Calot's triangle HU > 30, senior surgeons are recommended, with backup plans for conversion to open surgery (including subtotal cholecystectomy or fundus-first technique) or adhesion - separation equipment. Surgical timing optimization: The model helps determine the PTGBD- to -LC “window”. For instance, in patients with persistently elevated CRP (> 8.2 mg/L) despite PTGBD, extending the interval to allow for resolution of systemic inflammation ‒ through continued antibiotic therapy and close clinical monitoring ‒ may be considered before proceeding with LC. Conversely, in patients with severe local adhesions (GDAI > 0.7, Calot's triangle HU > 30) but normalized inflammatory markers, early surgery is advisable to prevent progression of fibrotic changes that could further complicate dissection. The median PTGBD-to-LC interval in our study (52-days overall) was shorter than that reported by Inoue et al. (97-days)^25^ and Ebinuma et al. (73-days).^26^ This discrepancy may be attributed to differences in institutional protocols, healthcare system logistics, patient selection criteria, or definitions of the interval (e.g., from PTGBD to LC vs. from discharge to LC). Additionally, variations in the severity of cholecystitis and the threshold for performing LC could influence timing decisions. Our findings underscore that optimal timing should not be based solely on a fixed interval but on a combination of clinical, laboratory, and imaging factors. We emphasize that the optimal timing for LC after PTGBD should not be determined by a fixed time interval alone. Instead, it should be individualized based on a comprehensive assessment of clinical status (resolution of symptoms), laboratory parameters (normalization of inflammatory markers such as CRP), and imaging findings (regression of pericholecystic fluid, stabilization of adhesions). Our model supports this multidimensional approach by providing objective criteria to guide timing decisions.

Preoperative risk stratification using this model may help reduce unplanned conversion rates if applied prospectively to guide surgical planning, such as assigning high-risk patients to experienced surgeons and preparing appropriate equipment. The CRP cut-off used in this study should be interpreted as a model-based stratification threshold rather than a strict clinical decision limit; modest CRP elevations alone do not imply surgical difficulty, but reflect residual systemic inflammation that gains predictive relevance only when combined with local anatomical and imaging findings in the multivariable model. However, this study has limitations. As a single-center retrospective study, it doesn't account for subjective factors like surgeon experience, which senior surgeons might adjust to reduce difficulty. No universal PTGBD-LC difficulty standard exists internationally. Our difficult LC thresholds (operative time > 110-min, blood loss > 150 mL, or conversion) were adapted from established LC difficulty literature^15^, ^16^, ^17^, ^18^ and aligned with TG18 operative outcome parameters. Lastly, psychological assessment relies solely on the HADS; future research could combine it with stress-hormone tests like cortisol to better explore the mechanisms. It should be emphasized that “non-difficult” surgery in this study refers to cases not meeting the TG18-defined objective thresholds and does not imply technical simplicity; rather, it reflects relative surgical difficulty within a cohort of patients who all underwent LC after PTGBD, a procedure inherently associated with increased operative complexity.

## Conclusions

Difficult LC after PTGBD arises from chronic inflammation-induced anatomical disruption. Preoperative CRP elevation, PCF, GDAI > 0.7, and Calot's triangle HU > 30 are independent predictors. Our nomogram provides excellent discrimination (AUC > 0.9) for identifying high-risk patients, potentially optimizing resource allocation and guiding surgical planning. Prospective multicenter validation is warranted.

## Ethics approval

This study was approved by the Ethics Committee of The Second Affiliated Hospital of Bengbu Medical University ([2026]KY044). Written informed consent was obtained from all patients for the publication of any potentially identifiable images or data included in this article.

## Authors’ contributions

Chaoyi-Zhou: Writing-review & editing; writing-original draft; visualization; software; methodology; investigation; formal analysis; conceptualization.

Liang-Chu: Visualization; software; methodology; investigation; formal analysis; data curation.

Zeliang-Xia: Writing-review & editing; supervision; resources; project administration; funding acquisition; conceptualization.

Zhaolong-Xu, and Zhengmin-Chen: Writing-review & editing; supervision; project administration; conceptualization.

## Funding

This work was funded by the 2023 Annual Science and Technology Project (Key Natural Science) of Bengbu Medical University (2023byzd112): Analysis of Risk Factors for Hyperlipidemic Acute Pancreatitis and Establishment and Validation of a Nomogram Risk Prediction Model.

## Declaration of competing interest

The authors declare that they have no known competing financial interests or personal relationships that could have appeared to influence the work reported in this paper.

## Data Availability

Data availability: Data will be made available on request.
